# Supplementary material for: Prediction of DNA Methylation based on Multi-dimensional feature encoding and double convolutional fully connected convolutional neural network
Source: PLoS Comput Biol. 2023 Aug 28;19(8):e1011370. doi: 10.1371/journal.pcbi.1011370 (PMC10461834; doi:10.1371/journal.pcbi.1011370)
Supplement: S1 Table — (DOCX) [file pcbi.1011370.s001.docx]

**S1 Table The results of MEDCNN prediction for independent datasets 5hmC,4mC and 6mA**

| Dataset | SN | SP | ACC | MCC | AUC |
| --- | --- | --- | --- | --- | --- |
| 5hmC_H.sapiens | 0.9845 | 0.8783 | 0.9368 | 0.8749 | 0.9226 |
| 5hmC_M.musculus | 0.9715 | 0.9702 | 0.9709 | 0.9417 | 0.9696 |
| 4mC_C.equisetifolia  4mC_F.vesca | 0.7727 | 0.7247 | 0.7487 | 0.4980 | 0.7657 |
| 4mC_F.vesca | 0.8005 | 0.8710 | 0.8396 | 0.6745 | 0.8361 |
| 4mC_S.cerevisiae  4mC_Tolypocladium | 0.7222 | 0.7551 | 0.7386 | 0.4775 | 0.7514 |
| 4mC_Tolypocladium | 0.7090 | 0.7761 | 0.7425 | 0.4861 | 0.7393 |
| 6mA_C.equisetifolia | 0.6559 | 0.8021 | 0.7457 | 0.4605 | 0.7303 |
| 6mA_D.melanogaster | 0.9049 | 0.9151 | 0.9100 | 0.8201 | 0.9109 |
| 6mA_R.chinensis | 0.8986 | 0.8377 | 0.8675 | 0.7370 | 0.8701 |
| 6mA_Xoc BLS256 | 0.8689 | 0.8688 | 0.8688 | 0.7377 | 0.8661 |
| 6mA_Tolypocladium | 0.8144 | 0.7537 | 0.7756 | 0.5484 | 0.7895 |
| 6mA_C.elegans | 0.9314 | 0.9063 | 0.9189 | 0.8380 | 0.9212 |
| 6mA_F.vesca | 0.9421 | 0.9450 | 0.9435 | 0.8871 | 0.9447 |
| 6mA_H.sapiens | 0.8913 | 0.9158 | 0.9035 | 0.8073 | 0.9049 |
| 6mA_S.cerevisiae | 0.8021 | 0.871053 | 0.8366 | 0.6748 | 0.8391 |
| 6mA_T.thermophile | 0.895 | 0.8975 | 0.8962 | 0.7925 | 0.8945 |
| 6mA_A.thaliana | 0.8552 | 0.8666 | 0.8609 | 0.7218 | 0.8611 |
