# Supplementary material for: Prediction of DNA Methylation based on Multi-dimensional feature encoding and double convolutional fully connected convolutional neural network
Source: PLoS Comput Biol. 2023 Aug 28;19(8):e1011370. doi: 10.1371/journal.pcbi.1011370 (PMC10461834; doi:10.1371/journal.pcbi.1011370)
Supplement: S2 Table — (DOCX) [file pcbi.1011370.s002.docx]

**S2 Table The results of different coding methods to predict the independent datasets 5hmC,4mC and 6mA.**

| Modification type | Dataset | Encode | SN | SP | ACC | MCC | AUC |
| --- | --- | --- | --- | --- | --- | --- | --- |
| 5hmC | H.sapiens | BPF | 0.9627 | 0.8859 | 0.9282 | 0.8557 | 0.9231 |
|  |  | NCP | 0.9752 | 0.8783 | 0.9216 | 0.8637 | 0.9217 |
|  |  | DPCP | 0.9752 | 0.8289 | 0.9094 | 0.8215 | 0.9123 |
|  |  | MEDCNN | 0.9845 | 0.8783 | 0.9370 | 0.8749 | 0.9226 |
|  | M.musculus | BPF | 0.9715 | 0.9673 | 0.9625 | 0.9389 | 0.9655 |
|  |  | NCP | 0.9702 | 0.9702 | 0.9652 | 0.9403 | 0.9741 |
|  |  | DPCP | 0.9634 | 0.9509 | 0.9575 | 0.9147 | 0.9580 |
|  |  | MEDCNN | 0.9715 | 0.9702 | 0.9710 | 0.9417 | 0.9696 |
| 4mC | C.equisetifolia | BPF | 0.7904 | 0.6894 | 0.7299 | 0.4823 | 0.7420 |
|  |  | NCP | 0.7778 | 0.6616 | 0.7197 | 0.4424 | 0.7239 |
|  |  | DPCP | 0.6970 | 0.5682 | 0.6326 | 0.2674 | 0.6359 |
|  |  | MEDCNN | 0.7727 | 0.7247 | 0.7490 | 0.4980 | 0.7657 |
|  | F.vesca | BPF | 0.8336 | 0.8341 | 0.8249 | 0.6655 | 0.8360 |
|  |  | NCP | 0.8229 | 0.8397 | 0.8262 | 0.6613 | 0.8317 |
|  |  | DPCP | 0.8283 | 0.8453 | 0.8307 | 0.6723 | 0.8425 |
|  |  | MEDCNN | 0.8005 | 0.8710 | 0.8400 | 0.6745 | 0.8361 |
|  | S.cerevisiae | BPF | 0.7525 | 0.7197 | 0.7261 | 0.4725 | 0.7437 |
|  |  | NCP | 0.7753 | 0.6566 | 0.7159 | 0.4349 | 0.7283 |
|  |  | DPCP | 0.6641 | 0.5758 | 0.6200 | 0.2408 | 0.6101 |
|  |  | MEDCNN | 0.7222 | 0.7551 | 0.7390 | 0.4775 | 0.7514 |
|  | Tolypocladium | BPF | 0.7264 | 0.7553 | 0.7280 | 0.4819 | 0.7400 |
|  |  | NCP | 0.6940 | 0.7770 | 0.7300 | 0.4730 | 0.7340 |
|  |  | DPCP | 0.6941 | 0.7880 | 0.7321 | 0.4842 | 0.7358 |
|  |  | MEDCNN | 0.7090 | 0.7761 | 0.7425 | 0.4861 | 0.7393 |
| 6mA | C.equisetifolia | BPF | 0.7365 | 0.6998 | 0.7139 | 0.4255 | 0.7215 |
|  |  | NCP | 0.7237 | 0.7102 | 0.7154 | 0.4239 | 0.7249 |
|  |  | DPCP | 0.7227 | 0.6769 | 0.6946 | 0.3894 | 0.6986 |
|  |  | MEDCNN | 0.6559 | 0.8021 | 0.7460 | 0.4605 | 0.7303 |
|  | D.melanogaster | BPF | 0.9041 | 0.9142 | 0.9091 | 0.8183 | 0.9105 |
|  |  | NCP | 0.9017 | 0.9166 | 0.9213 | 0.8184 | 0.9113 |
|  |  | DPCP | 0.9005 | 0.8892 | 0.8948 | 0.7897 | 0.8942 |
|  |  | MEDCNN | 0.9049 | 0.9151 | 0.9100 | 0.8201 | 0.9109 |
|  | R.chinensis | BPF | 0.8649 | 0.8377 | 0.8510 | 0.7025 | 0.8671 |
|  |  | NCP | 0.8851 | 0.8117 | 0.8477 | 0.6979 | 0.8557 |
|  |  | DPCP | 0.8243 | 0.7013 | 0.7616 | 0.5289 | 0.7403 |
|  |  | MEDCNN | 0.8986 | 0.8377 | 0.8680 | 0.7370 | 0.8701 |
|  | Xoc BLS256 | BPF | 0.8449 | 0.8722 | 0.8585 | 0.7173 | 0.8580 |
|  |  | NCP | 0.8391 | 0.8880 | 0.8635 | 0.7280 | 0.8666 |
|  |  | DPCP | 0.8477 | 0.8805 | 0.8701 | 0.7286 | 0.8636 |
|  |  | MEDCNN | 0.8689 | 0.8688 | 0.8690 | 0.7377 | 0.8661 |
|  | Tolypocladium | BPF | 0.8409 | 0.7163 | 0.7613 | 0.5355 | 0.7833 |
|  |  | NCP | 0.7973 | 0.7355 | 0.7579 | 0.5138 | 0.7642 |
|  |  | DPCP | 0.7557 | 0.7302 | 0.7394 | 0.4696 | 0.7464 |
|  |  | MEDCNN | 0.8144 | 0.7537 | 0.7760 | 0.5484 | 0.7895 |
|  | C.elegans | BPF | 0.9409 | 0.8786 | 0.9098 | 0.8211 | 0.9114 |
|  |  | NCP | 0.9126 | 0.8994 | 0.9060 | 0.8120 | 0.9072 |
|  |  | DPCP | 0.9075 | 0.8855 | 0.8965 | 0.7933 | 0.8941 |
|  |  | MEDCNN | 0.9314 | 0.9063 | 0.9190 | 0.8380 | 0.9212 |
|  | F.vesca | BPF | 0.9624 | 0.9009 | 0.9318 | 0.8651 | 0.9331 |
|  |  | NCP | 0.9468 | 0.9088 | 0.9278 | 0.8563 | 0.9262 |
|  |  | DPCP | 0.9405 | 0.9119 | 0.9263 | 0.8529 | 0.9238 |
|  |  | MEDCNN | 0.9421 | 0.9450 | 0.9440 | 0.8871 | 0.9447 |
|  | H.sapiens | BPF | 0.8902 | 0.9063 | 0.9119 | 0.7965 | 0.9013 |
|  |  | NCP | 0.8840 | 0.9177 | 0.9008 | 0.8021 | 0.9019 |
|  |  | DPCP | 0.8545 | 0.9074 | 0.8808 | 0.7628 | 0.8810 |
|  |  | MEDCNN | 0.8913 | 0.9158 | 0.9030 | 0.8073 | 0.9049 |
|  | S.cerevisiae | BPF | 0.8325 | 0.8316 | 0.8220 | 0.6640 | 0.8391 |
|  |  | NCP | 0.8615 | 0.7750 | 0.8182 | 0.6388 | 0.8202 |
|  |  | DPCP | 0.7348 | 0.8434 | 0.7892 | 0.5817 | 0.7886 |
|  |  | MEDCNN | 0.8021 | 0.8711 | 0.8370 | 0.6748 | 0.8391 |
|  | T.thermophile | BPF | 0.9119 | 0.8755 | 0.8737 | 0.7879 | 0.8954 |
|  |  | NCP | 0.9415 | 0.8730 | 0.9072 | 0.8164 | 0.9105 |
|  |  | DPCP | 0.9145 | 0.8818 | 0.8981 | 0.7967 | 0.8920 |
|  |  | MEDCNN | 0.8950 | 0.8975 | 0.8960 | 0.7925 | 0.8945 |
|  | A.thaliana | BPF | 0.8449 | 0.8728 | 0.8588 | 0.7179 | 0.8601 |
|  |  | NCP | 0.8505 | 0.8578 | 0.8541 | 0.7083 | 0.8561 |
|  |  | DPCP | 0.8469 | 0.8340 | 0.8404 | 0.6809 | 0.8398 |
|  |  | MFE | 0.8552 | 0.8666 | 0.8610 | 0.7218 | 0.8611 |
