# Supplementary material for: Prediction of DNA Methylation based on Multi-dimensional feature encoding and double convolutional fully connected convolutional neural network
Source: PLoS Comput Biol. 2023 Aug 28;19(8):e1011370. doi: 10.1371/journal.pcbi.1011370 (PMC10461834; doi:10.1371/journal.pcbi.1011370)
Supplement: S3 Table — (DOCX) [file pcbi.1011370.s003.docx]

**S3 Table The results of different models predicted for independent datasets 5hmC,4mC and 6mA.**

| Modification type | Dataset | Model | SN | SP | ACC | MCC | AUC |
| --- | --- | --- | --- | --- | --- | --- | --- |
| 5hmC | H.sapiens | iDNA-MS | 0.9770 | 0.9181 | 0.9475 | 0.8966 | 0.9620 |
|  |  | iDNA-AB | 0.9787 | 0.9181 | 0.9484 | 0.8984 | 0.9597 |
|  |  | MEDCNN | 0.9845 | 0.8783 | 0.9368 | 0.8749 | 0.9226 |
|  |  | iDNA-ABT | 0.9863 | 0.9121 | 0.9492 | 0.9009 | 0.9553 |
|  | M.musculus | iDNA-MS | 0.9685 | 0.9668 | 0.9676 | 0.9353 | 0.9835 |
|  |  | iDNA-AB | 0.9587 | 0.9668 | 0.9628 | 0.9255 | 0.9746 |
|  |  | MEDCNN | 0.9715 | 0.9702 | 0.9709 | 0.9417 | 0.9696 |
|  |  | iDNA-ABT | 0.9706 | 0.9663 | 0.9685 | 0.9369 | 0.9757 |
| 4mC | C.equisetifolia | iDNA-MS | 0.7275 | 0.7248 | 0.7109 | 0.4520 | 0.7900 |
|  |  | iDNA-AB | 0.5574 | 0.8634 | 0.7104 | 0.4420 | 0.7825 |
|  |  | MEDCNN | 0.7727 | 0.7247 | 0.7487 | 0.4980 | 0.7657 |
|  |  | iDNA-ABT | 0.7923 | 0.8579 | 0.8251 | 0.6517 | 0.8555 |
|  | F.vesca | iDNA-MS | 0.8229 | 0.8205 | 0.8217 | 0.6433 | 0.8991 |
|  |  | iDNA-AB | 0.8653 | 0.8153 | 0.8403 | 0.6814 | 0.9116 |
|  |  | MEDCNN | 0.8005 | 0.8710 | 0.8396 | 0.6745 | 0.8361 |
|  |  | iDNA-ABT | 0.8520 | 0.8321 | 0.8420 | 0.6842 | 0.9070 |
|  | S.cerevisiae | iDNA-MS | 0.6380 | 0.7543 | 0.6962 | 0.3950 | 0.7612 |
|  |  | iDNA-AB | 0.7128 | 0.6694 | 0.6911 | 0.3826 | 0.7355 |
|  |  | MEDCNN | 0.7222 | 0.7551 | 0.7386 | 0.4775 | 0.7514 |
|  |  | iDNA-ABT | 0.6694 | 0.7361 | 0.7027 | 0.4064 | 0.7537 |
|  | Tolypocladium | iDNA-MS | 0.6940 | 0.7250 | 0.7095 | 0.4192 | 0.7804 |
|  |  | iDNA-AB | 0.7156 | 0.7552 | 0.7354 | 0.4712 | 0.8045 |
|  |  | MEDCNN | 0.7090 | 0.7761 | 0.7425 | 0.4861 | 0.7393 |
|  |  | iDNA-ABT | 0.7216 | 0.7549 | 0.7383 | 0.4768 | 0.8057 |
| 6mA | C.equisetifolia | iDNA-MS | 0.6769 | 0.7540 | 0.7155 | 0.4322 | 0.7848 |
|  |  | iDNA-AB | 0.7029 | 0.7465 | 0.7247 | 0.4498 | 0.7848 |
|  |  | MEDCNN | 0.6559 | 0.8021 | 0.7457 | 0.4605 | 0.7303 |
|  |  | iDNA-ABT | 0.6891 | 0.7765 | 0.7328 | 0.4673 | 0.7902 |
|  | D.melanogaster | iDNA-MS | 0.9060 | 0.8994 | 0.8962 | 0.8050 | 0.9620 |
|  |  | iDNA-AB | 0.8881 | 0.9183 | 0.9032 | 0.8068 | 0.9585 |
|  |  | MEDCNN | 0.9049 | 0.9151 | 0.9100 | 0.8201 | 0.9109 |
|  |  | iDNA-ABT | 0.9038 | 0.9205 | 0.9122 | 0.8244 | 0.9544 |
|  | R.chinensis | iDNA-MS | 0.8400 | 0.7933 | 0.8167 | 0.6340 | 0.9020 |
|  |  | iDNA-AB | 0.8261 | 0.7291 | 0.7776 | 0.5578 | 0.8373 |
|  |  | MEDCNN | 0.8986 | 0.8377 | 0.8675 | 0.7370 | 0.8701 |
|  |  | iDNA-ABT | 0.8094 | 0.8428 | 0.8261 | 0.6525 | 0.8789 |
|  | Xoc BLS256 | iDNA-MS | 0.8050 | 0.8867 | 0.8449 | 0.6390 | 0.9251 |
|  |  | iDNA-AB | 0.8707 | 0.8558 | 0.8633 | 0.7266 | 0.9332 |
|  |  | MEDCNN | 0.8689 | 0.8688 | 0.8688 | 0.7377 | 0.8661 |
|  |  | iDNA-ABT | 0.8890 | 0.8492 | 0.8694 | 0.7394 | 0.9261 |
|  | Tolypocladium | iDNA-MS | 0.7389 | 0.7768 | 0.7342 | 0.5161 | 0.8211 |
|  |  | iDNA-AB | 0.7188 | 0.8105 | 0.7647 | 0.5316 | 0.8289 |
|  |  | MEDCNN | 0.8144 | 0.7537 | 0.7757 | 0.5484 | 0.7895 |
|  |  | iDNA-ABT | 0.7176 | 0.8301 | 0.7738 | 0.5512 | 0.8361 |
|  | C.elegans | iDNA-MS | 0.8450 | 0.8648 | 0.8537 | 0.7099 | 0.9311 |
|  |  | iDNA-AB | 0.8719 | 0.8807 | 0.8763 | 0.7525 | 0.9408 |
|  |  | MEDCNN | 0.9314 | 0.9063 | 0.9189 | 0.8380 | 0.9212 |
|  |  | iDNA-ABT | 0.8817 | 0.8990 | 0.8903 | 0.7808 | 0.9433 |
|  | F.vesca | iDNA-MS | 0.9239 | 0.9233 | 0.9226 | 0.8472 | 0.9759 |
|  |  | iDNA-AB | 0.9220 | 0.9246 | 0.9233 | 0.8466 | 0.9674 |
|  |  | MEDCNN | 0.9421 | 0.9450 | 0.9435 | 0.8871 | 0.9447 |
|  |  | iDNA-ABT | 0.9233 | 0.9304 | 0.9268 | 0.8244 | 0.9544 |
|  | H.sapiens | iDNA-MS | 0.8396 | 0.9201 | 0.8799 | 0.7623 | 0.9507 |
|  |  | iDNA-AB | 0.8814 | 0.8867 | 0.8840 | 0.7681 | 0.9511 |
|  |  | MEDCNN | 0.8913 | 0.9158 | 0.9035 | 0.8073 | 0.9049 |
|  |  | iDNA-ABT | 0.8940 | 0.9020 | 0.8980 | 0.7960 | 0.9510 |
|  | S.cerevisiae | iDNA-MS | 0.7332 | 0.8442 | 0.7855 | 0.5810 | 0.8708 |
|  |  | iDNA-AB | 0.7676 | 0.8204 | 0.7940 | 0.5888 | 0.8620 |
|  |  | MEDCNN | 0.8021 | 0.8711 | 0.8366 | 0.6748 | 0.8391 |
|  |  | iDNA-ABT | 0.7237 | 0.8785 | 0.8011 | 0.6096 | 0.8709 |
|  | T.thermophile | iDNA-MS | 0.9567 | 0.7637 | 0.8563 | 0.7342 | 0.9260 |
|  |  | iDNA-AB | 0.9465 | 0.8011 | 0.8738 | 0.7556 | 0.9366 |
|  |  | MEDCNN | 0.8950 | 0.8975 | 0.8962 | 0.7925 | 0.8945 |
|  |  | iDNA-ABT | 0.9334 | 0.8154 | 0.8740 | 0.7540 | 0.9310 |
|  | A.thaliana | iDNA-MS | 0.7993 | 0.8687 | 0.8340 | 0.6697 | 0.9093 |
|  |  | iDNA-AB | 0.8372 | 0.8723 | 0.8548 | 0.7100 | 0.9239 |
|  |  | MEDCNN | 0.8552 | 0.8666 | 0.8609 | 0.7218 | 0.8611 |
|  |  | iDNA-ABT | 0.8233 | 0.8842 | 0.8538 | 0.7088 | 0.9184 |
