# Supplementary material for: Prediction of DNA Methylation based on Multi-dimensional feature encoding and double convolutional fully connected convolutional neural network
Source: PLoS Comput Biol. 2023 Aug 28;19(8):e1011370. doi: 10.1371/journal.pcbi.1011370 (PMC10461834; doi:10.1371/journal.pcbi.1011370)
Supplement: Attachment — Submitted filename: point to point responses.docx [file pcbi.1011370.s004.docx]

Dear Dr Piero Fariselli ,Dr Sushmita Roy and Reviewers:

Thank you for your comments concerning our manuscript (Manuscript ID: PCOMPBIOL-D-23-00775). Those comments are all valuable and very helpful for revising and improving our paper. We have studied comments carefully and have made correction which we hope meet with approval. Revised portion are marked in red in the revised paper. Please see our point to point responses below, the original comments are in black, and our responses are in blue.

Response to Reviewer 1 Comments

**Point 1.** In the abstract, the authors mention that "most models have been built in terms of of a single methylation type. To address the above-mentioned issues, a deep learning-based method was proposed in this study for DNA methylation site prediction, termed the MEDCNN model.". However, based on the provided description, it seems that also the MEDCNN model has actually been trained separately on different species to predict a single methylation type. Please clarify this aspect for better understanding.

**Response 1:** Thank you for the kind comment. Indeed, as you mentioned, I did not provide a clear enough description. The MEDCNN model was trained on different species for each of the three methylation types, and our objective was to develop a generalized method for predicting methylation sites, rather than being restricted to a single methylation type. In the revised version, we have made further clarifications and included additional background information and explanations to improve readers' understanding of our work. We believe these revisions enhance the clarity and readability of the paper. Thank you again for your valuable comments.

**Point 2.** The dataset described in Table 1 appears to differ from the one used by Lv. et al. for training iDNA-MS. Notably, the dataset employed in this study includes a greater amount of training and testing data compared to Lv. et al. Moreover, it remains unclear how the training and testing data were generated. Could you please provide more details regarding the dataset acquisition process and the methodology used to partition the data into training and testing sets? Specifically, does the dataset exhibit any sequence similarity between the training and testing sequences?

**Response 2:** Thank you for your response. In this paper, we utilized the datasets provided by Lv et al. However, we reorganized the dataset due to an observation made after acquiring it from Lv et al. We found that the training set and the test set had a nearly equal ratio of 1:1. To address this, we merged the training set and the test set for each species individually. For datasets with a large number of gene sequences, we redistributed them into training and test sets using a 7:3 ratio. For datasets with relatively small amounts of data, we divided them using an 8:2 ratio. It is important to note that in the previous work of Lv et al., they employed the CD-HIT procedure to remove sequences with over 80% sequence similarity. Therefore, the dataset we used did not exhibit sequence similarity. We have provided further explanations regarding these modifications in the revised version, particularly in the section dedicated to datasets presentation. Thank you for your valuable comments and feedback.

**Point 3. The limited description of the training/testing data raises concerns regarding the comparison results presented in the section titled "Experimental results compared with other models." Several points deserve clarification:**

**- Is it possible that the prediction performance of MEDCNN is influenced by the larger training dataset used?**

**- To ensure a robust comparison with iDNA-MS, it would be better to use precisely its training and testing data. For instance, could there be an overlap between the test set used here and training set of iDNA-MS?**

**- Overall, figures 6 and 7 appear to exhibit comparable results, without any discernible predictor outperforming the other. It is advisable to conduct statistical significance tests, such as t-tests or Wilcoxon tests, to determine whether there are statistically significant differences in prediction performance.**

**Response 3:** Thank you for this helpful comment. The prediction performance of the model is indeed influenced by the size of the training dataset. In the case of some species, where the amount of available data is relatively small, the resulting prediction performance may be unsatisfactory, as stated in the conclusion section. I appreciate your reminder on this point.

In the model performance comparison experiments, we utilized the same dataset, as mentioned in the article. For the results comparison part, it is true that there is no significant difference in some of the performance comparisons, as you said.To address this concern, we conducted the Wilcoxon test in the revised version to statistically evaluate and compare the significant differences between the models. Please refer to the revised section on experimental results for a detailed description of the comparison with other models.

Thank you for providing valuable comments, which have helped improve the clarity and rigor of our paper.

**Point 4.** I have some concerns regarding the effectiveness of the NCP features utilized in the model. Do these features genuinely contribute to improved performance? They seem akin, albeit not formally, to a one-hot encoding of the four nucleotides. It is reasonable to assume that a machine learning approach would readily learn a mapping between BFP and NCP, as it represents a one-to-one mapping between binary vectors. Additionally, there appears to be little discrepancy in performance between these two features when used individually, as demonstrated in the "Experimental results of different feature encoding" section. Kindly provide further justification for the necessity of these features. Also in this case, employing a statistical significance test could aid in elucidating whether certain features or their combinations outperform others.

**Response 4:** Thank you for the details. We fully understand your concerns, and in the revised version, we have addressed them by including citations to several articles that validate the validity and effectiveness of NCP coding. For instance, we have cited the article：

Wei L, He W, Malik A, Su R, Cui L, Manavalan B. Computational prediction and interpretation of cell-specific replication origin sites from multiple eukaryotes by exploiting stacking framework. Briefings in Bioinformatics. 2021;22(4):bbaa275.

which utilizes both NCP coding and BPF coding. The article has conducted experiments comparing the performance of these coding methods, and the results demonstrate that these features do improve the performance of machine learning methods.

To determine whether certain features or their combinations outperform others, we have carefully considered and revised them accordingly. We have re-analyzed the relevant data and performed Wilcoxon tests to statistically evaluate the significance of the differences. We believe that these revisions adequately address this issue and enhance the overall credibility of the paper. For detailed information regarding these revisions, please refer to section’ Experimental results of different feature encoding’.

Thank you for providing valuable feedback, which has greatly contributed to strengthening the validity and effectiveness of our research. **Point 5.** Regarding the section titled "Experimental results of cross-species validation," the concluding statement appears unsupported by the experimental tests depicted in Figure 5 since the authors' assertion appears valid solely for C. equisetifolia.

**Response 5:**

Thank you for your clarification. The experimental section was designed to showcase that MEDCNN models can achieve favorable prediction results for different species within the same methylation type. It is not limited to just C.equisetifolia., and we have provided additional details in the revised version to underscore the validity of this perspective. We appreciate your input and have taken it into account to enhance the clarity and emphasis of our viewpoint. Thank you again for your valuable feedback.

**Point 6.** **The manuscript would benefit from a thorough proofreading to rectify typos. I have compiled a list of some identified errors:**

**- Page 9, Equation 6: The term "D_i" lacks an explanation. Also, it has been possibly mistyped as "D_I".**

**- Page 9, line 182: The sentence beginning with "However, since a 2D feature ..." requires revision.**

**- Page 12, line 254: The meaning of "to identify 17 datasets" is unclear.**

**- Page 12, Figure 4(a): The labels BFP, NCP, and DPCP are probably a typo. If not, please clarify their meaning.**

**- Page 13, line 176: Add a period before "Five datasets."**

**- Page 13, line 279: Please revise the sentence starting with "predict whether the weather."**

**- Page 15, line 304: Please revise the sentence beginning with "iDNA-AB and iDNA-ABT, ..."**

**Response 6:** Thank you for your patience in checking, we did find quite a few minor errors in our careful comparison, and we have thoroughly proofread the manuscript and made corrections.

Finally, we once again express our sincere appreciation for the valuable suggestions you provided during the review process. Your expertise and insights are of great importance to our research work. We will continue to strive to improve our paper and enhance its quality and academic value under your guidance. If you have any further suggestions or comments, we would welcome and be happy to hear them. Thank you again for your time and dedication.

Response to Reviewer 2 Comments

**Point 1.** In Fig3 abcde, the authors illustrated the profiles or distributions for each measure, but the readers cannot understand what they mean by the profiles. Explain why the figures have profiles clearly. They should explain details in the figures intelligibly in the figure legends.

**Response 1:** Thank you for your valuable comments. We apologize for the lack of clear explanation regarding the profiles in the diagram, which may have made it difficult for the reader to understand. We have made the following modifications to address this issue:

1. Added explanatory notes: We have included additional notes in the diagram to clarify the meaning of the profiles.

2. Supplementary notes: In addition to the explanatory notes, we have incorporated supplementary notes. These notes provide further context and additional information to enhance the reader's understanding of the diagram.

By implementing these changes, we aim to improve the clarity and comprehensibility of the diagram, allowing the reader to gain a quick understanding of its meaning. We appreciate your feedback.

**Point 2.** The authors employed convolutional block attention module after the convolutional layer to gain insights into the important features and their locations along the channels and spatial axes by reasoning about their attention images. However, they did not show the attention map and did not explain the insights on the important features. Does the addition of the attention module contribute to the improvement in prediction performance?

**Response 2:** Thank you for your helpful comments, and as you suggested, we have added this section to the revised version to explain the convolutional block attention module in more detail. Thank you.

**Point 3.** **It may be hard to understand that MDECNN outperformed the existing methods, because the authors did not investigate the latest methods for the respective methylation site prediction in DNA.**

**Response 3:** We understand and appreciate your concern. We apologize for not providing more detailed information about the latest methods for specific methylation site prediction in our paper. Our focus has been primarily on developing a generic methylation site prediction method.

We have taken your feedback into consideration and made revisions to the section titled "Experimental Results Compared with Other Models." These revisions aim to provide a better understanding to the reader regarding why our model, MDECNN, outperforms other generic methylation site prediction methods. We have included additional explanations and relevant comparisons to highlight the strengths and advantages of our approach.

By addressing this concern, we believe that the revised section will provide more clarity and context, allowing the reader to gain a deeper understanding of the superior performance of our model compared to other existing generic methylation site prediction methods.

**Point 4.** https://github.com/gnnumsli/DNA-Methylation is immature. This URL site must be prepared so that the users can execute the program and obtain the same results as provided by the manuscript.

**Response 4:** Thank you for this useful comment. We value your evaluation and guidance of our research efforts. Regarding your comment that https://github.com/gnnumsli/DNA-Methylation is immature, we apologize. We have uploaded all the code and data parts on GitHub, and readers can download and run them by themselves, and we believe we can get the same experimental results as in the manuscript.

Thank you again for your time and dedication. If you have any other suggestions or comments, we are more than welcome and happy to hear them

We appreciate for your warm work earnestly, and hope that these revisions are satisfactory and that the revised version will be acceptable for publication.

Thank you very much for your work concerning my paper and wish you all the best.

Yours sincerely,

LI Mengshan

Email: msli@gnnu.edu.cn
